# The effect of personality traits on consumer behaviour among football fans: The mediating role of fan loyalty

**DOI:** 10.1371/journal.pone.0343795

**Published:** 2026-02-23

**Authors:** Hakan Akgül, Can Özgider, İlhan Adiloğulları

**Affiliations:** Department of Sports Management, Faculty of Sport Sciences, Çanakkale Onsekiz Mart University, Çanakkale, Türkiye; Mugla Sitki Kocman University: Mugla Sitki Kocman Universitesi, TÜRKIYE

## Abstract

The aim of the current study was to examine the impact of personality traits on consumer behaviour among football fans in a Turkish setting by focusing on the mediating role of sports fans’ loyalty. Drawing on the Social Identity Theory, this research examines how the Big Five Personality characteristics, including openness, agreeableness, conscientiousness, extraversion, and emotional stability, influence sports consumption through loyalty mechanisms. Data were gathered from 929 active university students who were all active football fans by using three validated scales for personality, fan loyalty, and consumption behaviour. The indirect effects of personality traits on consumer behaviour through fan loyalty were significant, whereas the direct effects were not statistically significant. Besides, the results indicate that personality traits are associated with sports fan consumption patterns through fan loyalty. This highlights fan loyalty as a potential mechanism linking individual characteristics with collective behaviour patterns. Furthermore, the findings shed light on our understanding of sports fan engagement by combining perspectives from personality theory and social identity theory. Ultimately, it offers practical implications for both sports marketers and club managers seeking ways to foster long-term relationships with their fans, particularly within collectivist cultures.

## Introduction

Due to evolving life conditions, sports have become a vital social and economic power [1]. Besides, its benefits for promoting health and social well-being have transformed into a global industry [2,3]. Football is the most popular sport, because it has been attracting millions of sports fans globally, and therefore plays an essential role in the sports economy [4]. Mega football events like the FIFA World Cup and UEFA Champions League can serve not only as entertainment but also as global commercial organisations and can also impact cultural identity [5,6]. This commercial expansion has a strong association with sports fan engagement. Since sports fans are the buyers of sports-related products, sports clubs would struggle to sustain their income without the support of the fans [7,8]. Nowadays, successful sports clubs primarily rely on their teams' performance and ability to build long-lasting relationships with sports fans. As a result, research on sports management highlights understanding the fan loyalty and consumer behaviour [9,10].

Sports fans have a strong and lasting attachment to their favourite team even if they lose [11,12]. Loyal fans are more likely to take part in consumption activities, including purchasing seasonal tickets, merchandise, and attending games [13,14]. Research implies that loyalty has been seen as a significant factor influencing consumer behaviour in sports [15–17]. However, the patterns of loyalty in sports fans may change. In general, fans are categorized as local, loyal, temporary, and dysfunctional, each of them revealing distinct ways to identify themselves [18,19]. Being able to understand these differences is crucial to tailor marketing strategies. Accordingly, emotional and identity factors can substantially influence fans’ behaviour. These kinds of dense emotions throughout football games can lead fans to experience extreme feelings, ranging from joy to frustration, which can substantially have an impact on fans’ decisions to attend games, buy merchandise of their favourite teams [20,21]. Indeed, having sports clubs’ merchandise is the symbol of belonging, which can help to foster identity and attachment to their clubs in a visible way [22]. From the perspective of marketing, emotions and loyalty are vital for sports clubs to maintain a long-term engagement with their fans [23].

On the other hand, personality traits have also been studied as possible factor influencing sports fans’ behaviour. Social Identity Theory (SIT) states that involving in social groups can help individuals to gain a part of their self-identity [24]. Moreover, most sports fans can develop a strong relationship with their favourite team during their time in sports settings, even in difficult situations; this relationship may enhance loyalty [25]. In other words, sports fans who exhibit extroverted and conscientious characteristics are more likely to express their social identity through supporting their team and consuming their favourite team’s merchandise. Indeed, loyalty can help to reflect social identity and alter an individual’s preferences into attachment. Consequently, may lead to stable consumption patterns such as attending games and purchasing licensed products [26].

To understand the preferences of the consumers and their decision-making mechanisms, the Five-Factor Model, which encompasses openness, conscientiousness, agreeableness, extraversion, and emotional stability, is frequently used [27–29]. Attending matches for socialisation is related to extraversion, searching for new experiences is associated with openness, and maintaining this behaviour in sports is associated with conscientiousness [30,31]. Furthermore, it has been indicated that personality traits have a strong influence on how loyalty is exhibited with attachment and purchasing behaviour [32,33]. Yet, it has been reported that there are some discrepancies in the literature, some studies implying the predictive role of personality [34,35], others underscoring the importance of loyalty as the main factor, which can lead to consumption behaviour, without considering the role of personality traits [36,37]. This ongoing discussion suggests that further research is necessary among different cultures.

Accordingly, in order to explore personality traits and loyalty of the sports fans, the Turkish setting appears to offer a very vibrant context. Apart from its popularity, football in Türkiye is very important cultural symbol harmonised with local ties, social identity and family traditions [38–40]. Indeed, the Turkish sports fans are well-known for their devotion, readiness, and strong emotional connection. Although sports clubs in Türkiye mainly rely on their loyal fans for revenue, they often face issues with fluctuating attendance and financial uncertainty [41–43]. Most of the research carried out has been focused on Western populations, in which consumption routines notably vary from those of other cultures, such as Türkiye, highlighting the expression of emotions and collectivist behaviour. Furthermore, the mediating role of sports fan loyalty in the Turkish context is not well understood in non-Western cultures. In response, the present research aimed to investigate how football fans’ loyalty influences the relationship between consumption habits and personality traits.

Despite an extensive body of research on sport consumption, fan loyalty, and personality traits primarily among Western populations, it remains uncertain whether personality traits directly drive consumption behaviour or do so mainly through mediating psychological mechanisms. While SIT is often referenced in sport fandom studies, it is rarely incorporated into empirical models. This research examines fan loyalty as a key mediating factor connecting Big Five personality traits to football fan consumption behaviour in Türkiye, considering the cultural context to explore how collectivist identification influences sports consumption.

Guided by SIT, this study expects that personality traits are positively associated with fan loyalty, which in turn influences the consumption behaviour of football fans. Considering the mixed results of earlier research and Türkiye’s collectivist culture, the direct effects of personality traits are expected to be weaker than the indirect effects mediated through fan loyalty.

## Materials and methods

### Research design

**Fig 1:**
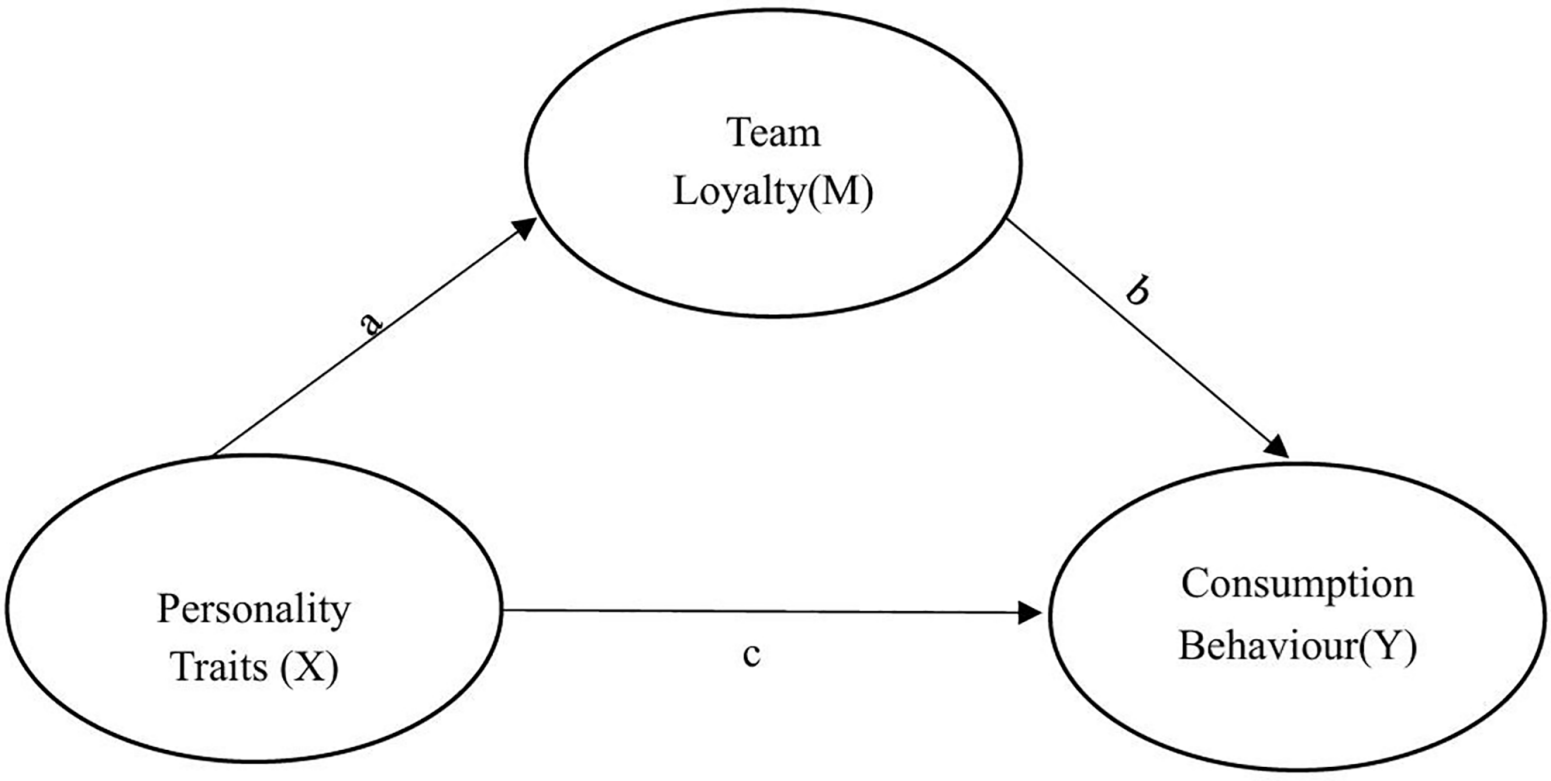
The mediating role of team loyalty in personality traits and consumption behaviour.

A correlational research design was used to explore the relationship between multiple variables [44] and to investigate how fan loyalty traits of the participants mediate the link between football fans’ personality traits and their consumption patterns. Hayes’s [45] mediation model analysis was applied to test it. This model analyses how X influences Y via the mediating variable (M). The effect is found by multiplying X’s impact on M (a) by M’s impact on Y (b). Hayes suggests testing the indirect effect directly and checking whether it varies significantly. Therefore, the existence of the mediating effect relies on whether the product a*b is substantial. This method avoids separate tests for each path, as in traditional mediation analysis, and focuses only on the significance of the indirect effect (a*b) [45] (Fig 1).

Although the analyses were conducted using Hayes’s conceptual framework for mediation logic, they were performed in IBM SPSS AMOS to estimate indirect effects within a structural equation modelling framework. This method enables testing of mediation paths aligned with Hayes’sapproach and uses AMOS’s bootstrapping techniques to derive confidence intervals for indirect effects.

IBM SPSS AMOS (Version 25) was utilised to test the mediating role of M. The analysis involved testing direct, and indirect effect scores to evaluate how M mediates the relationship between X and Y. The significance of these effects was tested using a 95% bootstrap confidence interval (5.000). The effect size was evaluated based on the magnitude of the standardised direct and indirect path coefficients. For mediation to be confirmed, the bootstrap confidence interval’s lower and upper bounds needed to be entirely above or below zero [46]. Separate mediation models were developed for each personality trait to minimise potential multicollinearity among the Big Five dimensions and to better understand each trait’s indirect effect via fan loyalty. Besides, although bootstrapping procedures improve the estimation of indirect effects, they do not resolve problems with temporal ordering or causal inference inherent in cross-sectional research designs.

### Population and sample recruitment

The participants were students at Çanakkale Onsekiz Mart University during the 2019–2020 academic year. The sample consisted of 929 university students, including 475 males and 454 females, all aged 18 or over. Since the study was conducted online and aimed to include all interested students, a convenience sampling method, a non-probability sampling technique, was used [47]. The inclusion criteria for participation in this research were (a) being an active student during the data collection, (b) being 18 years of age and older, and (c) agreeing to take part in the study voluntarily. Additionally, participants were required to identify themselves as active football fans who regularly follow football and their favourite team through attending or viewing games.

### Data collection process

The data were gathered online through a questionnaire distributed to students at Çanakkale Onsekiz Mart University via the University Information Management System (UBYS). The data collection period ran from 15 April 2020–12 October 2020. Before participating, students were informed about the research’s aim and confidentiality, and all gave written informed consent.

### Ethical considerations

Prior to collecting data, the ethics committee approval was gathered by the Institute of Social Sciences, Çanakkale Onsekiz Mart University, (Protocol No: 2020/16, Date: 5 March 2020). All procedures adhered to the ethical standards of the institutional research committee and followed the principles of the Declaration of Helsinki.

### Data collection tools

A Personal Information Form and three different scales, including the Fan Psychological Commitment Scale, the Ten-Item Personality Inventory (TIPI), and the Football Fan Consumption Behaviour Scale, were used to collect data. The Personal Information Form was designed to gather participants’ demographic profiles, like gender, age, marital status, faculty or school of study, educational level, residence type, funding source for education, favourite football team, and frequency of watching football matches.

### Fan Psychological Commitment Scale

The Fan Psychological Commitment Scale (FPCS), created by Matsuoka [48]. It evaluates the psychological loyalty of sports fans. Confirmatory factor analysis (CFA) revealed that the sub-dimensions of resource cost, social necessity, and psychological cost each have four items. Moreover, emotional commitment includes 5 items, personal identity 6, and regional commitment 7, for a total of 30 items across 6 sub-dimensions. It was adopted into Turkish by Bozgeyikli et al. [49]. They examined the scale’s validity and reliability in Turkish. The Turkish FPCS used CFA to evaluate construct validity and the factor structure. Although the factor structure did not precisely match the original, fit indices were similar to those of the original. The validity and reliability study conducted with university students suggests that the scale is suitable for comparable populations. The total scale achieved a Cronbach’s alpha of.96 in the present research, revealing excellent internal consistency. Although the original used a 7-point Likert scale, the Turkish version adapted it to a 5-point scale, ranging from (1) “Strongly Disagree” to (5) “Strongly Agree”.

### Ten-Item Personality Inventory (TIPI)

The Ten-Item Personality Inventory (TIPI), formed by Gosling et al. [50]. It evaluates the Big Five personality traits. It was adapted to Turkish by Atak [51]. The internal consistency (Cronbach’s α) for the sub-dimensions in this research was Openness to Experiences =  .83, Agreeableness = .81, Emotional Stability = .83, Conscientiousness = .84, and Extraversion = .86. Test–retest reliability with 54 participants showed acceptable stability over time: Openness = .89, Agreeableness = .87, Emotional Stability = .89, Conscientiousness = .87, and Extraversion = .88. Like the original, the Turkish TIPI includes ten items measuring five traits, openness to experience, extraversion, conscientiousness, emotional stability, and agreeableness, each represented by two items: Openness to Experiences (Items 5, 10), Extraversion (Items 1, 6), Conscientiousness (Items 3, 8), Emotional Stability (Items 9, 4), and Agreeableness (Items 7, 2). Responses are on a 7-point Likert scale, from 1 (Strongly Disagree) to 7 (Strongly Agree). Overall, the Turkish TIPI demonstrates acceptable reliability and validity.

### Football Fan Consumption Behaviour Scale

Football Fan Consumption Behaviour Scale (FFCBS), created by Kim [52] and adopted into Turkish culture by Köse [53]. The scale has 11 items and 4 sub-dimensions, including match attendance, media consumption, merchandise consumption, and word of mouth communication. All items are evaluated on a 5-point Likert scale. Besides, the scale’s overall internal consistency was.96, showing high reliability in the present study. The Cronbach’s alpha values from validity and reliability assessments indicate that the Turkish version of the scale is psychometrically sound and suitable for research into the consumption behaviours of football fans.

### Data analysis

The data were analysed by using IBM SPSS Statistics (Version 25), and descriptive statistics, including percentages and frequency distributions, were calculated. Prior to conducting inferential analyses, the normality and homogeneity of variance assumptions were tested and met. Relationships among variables were examined using Pearson’s correlation. Mediation models were tested through mediation analysis with IBM SPSS AMOS (Version 25). The significance level for all tests was set at *p* < .05.

## Results

Mediation analyses indicated a consistent pattern across all five personality traits. Fan loyalty had statistically significant indirect effects connecting personality traits to fan consumption behaviour, as indicated by bootstrapped confidence intervals that did not cross zero. However, direct effects of personality traits on consumption behaviour were not statistically significant in any models.

For openness to experience, the indirect effect of fan consumption behaviour through fan loyalty was statistically significant (*β* = 0.053, *SE* = 0.026, 95% CI [0.003, 0.105]). Nevertheless, the direct effect of openness to experience on consumption was not statistically significant (*β* = 0.017, *SE* = 0.017, 95% CI [−0.031, 0.034]) (Table 1).

**Table 1:** Mediating role of fan loyalty in the effect of openness to experience on consumer behaviour.

Type	*β*	*SE*	95% CI		*Z*	*p*
			Lower	Upper		
Indirect Effect	0.053	0.026	0.003	0.105	2.05	.040
Direct Effect	0.017	0.017	−0.031	0.034	0.10	.922

Agreeableness revealed a statistically significant indirect effect on consumption behaviour via fan loyalty (*β* = 0.204, *SE* = 0.036, 95% CI [0.135, 0.275]). The direct effect of agreeableness on consumption behaviour was not statistically significant (*β* = 0.004, *SE* = 0.025, 95% CI [−0.046, 0.053]) (Table 2).

**Table 2:** Mediating role of fan loyalty in the effect of agreeableness on consumer behaviour.

Type	*β*	*SE*	95% CI		*Z*	*p*
			Lower	Upper		
Indirect Effect	0.204	0.036	0.135	0.275	5.69	<.001
Direct Effect	0.004	0.025	−0.046	0.053	0.15	.880

The indirect effect of emotional stability on fan consumption behaviour through fan loyalty was statistically significant (*β* = 0.201, *SE* = 0.029, 95% CI [0.146, 0.256]). The direct effect of emotional stability on consumption behaviour was not statistically significant (*β* = 0.029, *SE* = 0.020, 95% CI [−0.010, 0.069]) (Table 3).

**Table 3:** Mediating role of fan loyalty in the effect of emotional stability on consumer behaviour.

Type	*β*	*SE*	95% CI		*Z*	*p*
			Lower	Upper		
Indirect Effect	0.201	0.029	0.146	0.256	7.04	<.001
Direct Effect	0.029	0.020	−0.010	0.069	1.45	.147

Conscientiousness showed a statistically significant indirect effect on fan consumption behaviour via fan loyalty (*β* = 0.208, *SE* = 0.033, 95% CI [0.143, 0.272]). The direct effect of conscientiousness on consumption behaviour was not statistically significant (*β* = −0.030, *SE* = 0.023, 95% CI [−0.073, 0.015]) (Table 4).

**Table 4:** Mediating role of fan loyalty in the effect of conscientiousness on consumer behaviour.

Type	*β*	*SE*	95% CI		*Z*	*p*
			Lower	Upper		
Indirect Effect	0.208	0.033	0.143	0.272	6.25	<.001
Direct Effect	−0.030	0.023	−0.073	0.015	−1.31	.191

For the extraversion, the indirect effect on fan consumption through fan loyalty was statistically significant (*β* = 0.104, *SE* = 0.026, 95% CI [0.053, 0.154]). The direct effect of extraversion on consumption behaviour was not statistically significant (*β* = −0.014, *SE* = 0.018, 95% CI [−0.048, 0.020]). (Table 5). Across all five mediation models, the indirect effects through fan loyalty were significant, whereas the direct effects were not significant.

**Table 5:** Mediating role of fan loyalty in the effect of extraversion on consumer behaviour.

Type	*β*	*SE*	95% CI		*Z*	*p*
			Lower	Upper		
Indirect Effect	0.104	0.026	0.053	0.154	4.03	<.001
Direct Effect	−0.014	0.018	−0.048	0.020	−0.80	.424

## Discussion

The present research examined the connection between personality traits and consumer behaviour among football fans, as well as the mediating role of fan loyalty in this connection. The findings indicate that the indirect effects of personality traits on consumer behaviour through fan loyalty were significant, whereas the direct effects were not. In other words, the impact of personality traits on fans’ consumption behaviour is indirect, mediated through loyalty. Given the cross-sectional design, the observed indirect effects should be understood as correlational rather than causal.

The current findings reveal that the link between personality traits and sports consumption behaviour is substantially influenced by cultural context. While research in Western populations generally reports direct links between personality traits such as extraversion, openness, and conscientiousness and sports consumption behaviours [25,27,34] no direct effects were found in the present research once fan loyalty was taken into account.

This difference may be interpreted through SIT [24] and the collectivist aspects of Turkish football culture, in which emotional bonds and group membership are considered important [38,39,42]. However, since this research did not measure cultural orientations like collectivism, any cultural interpretations should be considered theoretical. In this setting, personality traits appear to be indirectly connected to consumption behaviour through fan loyalty, suggesting fan loyalty may represent a culturally rooted link between personality traits and behavioural outcomes.

The findings are in line with SIT [24], which proposes that people may form connections with social groups, and these shape their behavioural patterns. Personality traits’ impact on team identification is a complex issue. The behavioural expression of identification may depend on the development of emotional loyalty. Whereas extroverted sports fans may be more prone to involvement with their favourite sports teams, tangible consumption behaviours like game attendance, buying licensed products, or media coverage following appear to be more closely associated with fan loyalty. This is inconsistent with the results of earlier studies [24,32,34], underscoring the mediating role of loyalty in fan behaviour.

In addition, these findings are different from some previous research [27,30,35], which implies that personality traits can directly impact consumer behaviour. The cultural context of the present research might explain this difference. Turkish football fandom is mainly characterised by collectivist tendencies, intense emotional bonds, and a sense of community belonging [38–40]. In this context, fan behaviour appears to be linked with group identity and a sense of belonging, and individual characteristics. Indeed, it has been stated that supporting a team in Türkiye is perceived as a form of social solidarity that transcends individual preference [41,42]. Therefore, while personality traits may reflect psychological tendencies, their association with behaviour seems to be linked to the level of emotional loyalty.

The results indicate a notable indirect association for each of the personality traits. For example, the indirect effect coefficients range from *β* = 0.053 (CI [0.003–0.105]) for openness to experience to *β* = 0.208 (CI [0.143–0.272]) for conscientiousness. The indirect impact of the extraversion is substantial (*β* = 0.104, *p* < .001); yet the direct impact is not (*β* = −0.014, *p* = .424). Because the mediation models were estimated separately, the magnitudes of the indirect effects were not statistically compared across traits and should be viewed as descriptive observations.

Furthermore, compatibility and emotional stability appear to be associated with more harmonious and enduring connections with favourite teams. On the other hand, fans who are very responsible and turn their loyalty into consistent, supportive actions. These patterns may indicate that loyalty serves as a psychological process connecting personality traits with group identity, rather than representing an outcome variable [23,33].

### Practical implications

The following practical implications are based on observed links between personality traits, fan loyalty and consumption behaviour. Accordingly, the findings suggest that especially for sports organisations seeking to improve fan loyalty and related consumption behaviours within the research context. Because personality characteristics showed indirect associations with consumption behaviour through loyalty, sports clubs may consider prioritising that support the development of fan loyalty. Boosting sports fans’ emotional loyalty and increasing their sense of community can support long-term engagement. Initiatives like developing fan clubs and regional projects can help sports fans establish their identity within a broader community. As it was proposed earlier, creating vibrant digital platforms such as social media can increase the loyalty of sports fans [7].

Since the fan culture has a collectivist nature in Türkiye, focusing on an identity-based marketing approach can have a significant impact. Moreover, highlighting the sports club’s history and its shared values by initiating campaigns can strengthen the sense of belonging of the sports fans. Additionally, offering programs, special events or exclusive content related to loyalty that rewards long-term support can foster the emotional bonds. In other words, focusing more on being in the community rather than personal characteristics can promote sustainable patterns in sports fans’ consumption.

From the perspective of sports managers, these findings suggest that initiatives aimed at improving fan loyalty could be linked to higher engagement results, like interest in season tickets and merchandise. Finally, these findings suggest that loyalty may play an essential psychological role in connecting social identity and personality with consumer behaviour. These practical implications should be considered in the research’s cross-sectional design and cultural context and may not apply directly to other environments.

### Limitations and future research

The present study has some limitations. Firstly, the sample is composed of 929 university students who were recruited online between April and October 2020, which can restrict the generalisability of the results. Moreover, the consumption tendencies of young, highly educated fans may differ from those of other age or socioeconomic groups. Future researchers can focus on comparative analyses with samples of different ages and income levels.

Secondly, the study’s cross-sectional nature did not allow for causal inferences. Longitudinal studies, however, can examine how loyalty forms over time and how personality traits affect this process. Furthermore, an experimental or intervention-based study can investigate the effects of strategies which was designed to boost loyalty on consumption.

Correlational and cross-sectional designs can restrict causal conclusions, so the mediation results should be viewed as associations rather than causations. While bootstrapping improves the estimation of indirect effects, it does not resolve issues of temporal order or prove causal pathways. Furthermore, reliance on convenience sampling limits the extent to which these findings can be generalized beyond the current sample. Thereby, the mediating role of fan loyalty observed in this research should be viewed as a correlational pattern, not as evidence of a causal psychological mechanism.

Ultimately, cultural context is a crucial factor in interpreting the study’s findings. Given the emotional and collectivist structure of Turkish football sports fan culture, it may be helpful to make cross-cultural comparisons with more individualist cultures to examine the universality of loyalty’s mediating role. Additionally, evaluating similar models on sports fans from different sports branches or with diverse fan profiles can improve the theoretical understanding of the link between personality, loyalty, and consumption behaviour.

## Conclusions

To conclude, the indirect effects of personality traits on consumer behaviour through fan loyalty were significant, whereas the direct effects were not significant. These findings highlight the relevance of emotional attachment and social identity as contextual factors that may shape sports consumption patterns, specifically within collectivist settings. However, given the cross-sectional design and the sample’s specific cultural characteristics, these findings should be interpreted cautiously. From both theoretical and managerial perspectives, strengthening fan loyalty may represent a potential process through which psychological engagement is linked to sustained behavioural economic attachment.
